# Synthesis of Cr(III)-Morin Complex: Characterization and Antioxidant Study

**DOI:** 10.1155/2014/845208

**Published:** 2014-01-29

**Authors:** Qadeer K. Panhwar, Shahabuddin Memon

**Affiliations:** Dr. M.A. Kazi Institute of Chemistry, National Center of Excellence in Analytical Chemistry, University of Sindh, Jamshoro 76080, Pakistan

## Abstract

The complex formation between Cr(III) and morin was carried out in methanol and confirmed by analytical characterization using UV-Vis, IR, ^1^H NMR, and TG-DTA. UV-Vis shows significant bathochromic shift in benzoyl upon coordination as well as IR well illustrates the peak shift of C=O group and formation of a O–Cr(III) bond. Likewise, ^1^H NMR studies clarify that Cr(III) metal ion replaces the 5OH proton hence; 5-hydroxy-4-keto site is employed by morin in chelation to form six-membered stable ring system out of three available chelating sites. In addition, TG-DTA denotes the presence of coordinated and crystalline water molecules. The melting point of the complex was found to be 389°C by DSC. In addition, Cr(III)-morin complex was found to be a more potent antioxidant than morin as evaluated by DPPH• and FRAP methods.

## 1. Introduction

Flavonoids have recently attracted great interest as potential therapeutic agents against a variety of diseases like those involving radical damage. These polyphenolic compounds, ubiquitous in higher plants, are commonly major dietary constituents. The biological and medicinal properties of flavonoids have been reviewed extensively, with wealth of data on their activity as reducing agents, hydrogen-donating antioxidants, and singlet oxygen quenchers [1–5]. They also show beneficial effects in age-associated diseases such as cardiovascular and cerebrovascular diseases, some forms of cancer, and Parkinson's and Alzheimer's diseases [6]. Many flavonoids are natural chelators and flavonoid metal complexes have showed significantly higher cytotoxic activity than those of the parent flavonoids. Besides, it is demonstrated that the coordination of metals like Cu(II) ion with bioactive ligands such as quercetin, morin, and chrysin can actually improve the pharmaceutical activity of the drugs themselves and reduce their toxicity effects [7].

**Figure 1:**
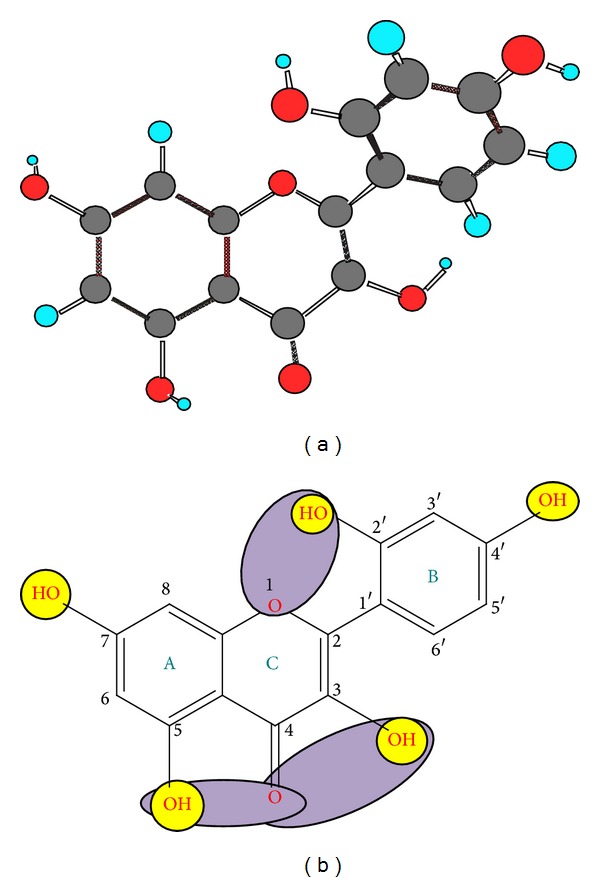
(a) The energy minimized chemical structure of morin; (b) yellow-labeled functional moieties indicate the antioxidant active centers, whereas violet-labeled functions indicate its multiple chelation sites.

Morin (3, 5, 7, 2′, 4′-pentahydroxyflavone; a yellowish pigment) is a bioflavonoid constituent of many herbs and fruits [8]. It is widely distributed in tea, coffee, cereal grains, and a variety of fruits and vegetables [9]. It has two aromatic rings (A and B) linked by an oxygen-containing heterocycle (ring C) shown in Figure 1. Morin is abundant in human diet and possesses potent antioxidant and metal ion chelating capacities and thus exerts various biological and biochemical effects including anti-inflammatory, antineoplastic, and cardioprotective activities [10]. As an antioxidant, it protects various human cells, like myocytes, endothelial cells, hepatocytes, and erythrocytes, against oxidative damages. Moreover, morin acts as a chemopreventive agent against oral carcinogenesis *in vitro* and *in vivo*. It is also reported that morin could modulate the activities of the metabolic enzymes, including cytochrome P450 [11]. Therefore, to save the humans from such diseases, morin must be added to the diet because it is sparingly soluble in water and consequently may limit its absorption and oral administration [12].

**Scheme 1:**
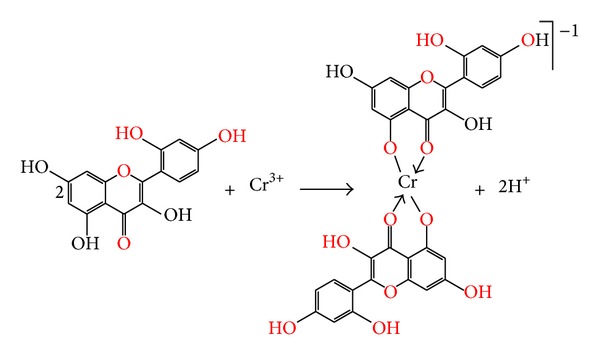
Proposed chemical reaction between Cr(III) metal ion and morin ligand molecule.

Chromium is widely used in many industries like electroplating, water cooling, pulp producing, tanning as well as ore, and petroleum refining processes. It exists in two stable oxidation states, that is, Cr(III) and Cr(VI). Cr(VI) is considered as more toxic relative to Cr(III). Thus, Cr (VI) exerts many harmful effects in humans; that is, it induces cancer and mutation in living cells, damages DNA-protein cross-links, and causes the single-strand breaks. On the other hand, Cr(III) is relatively less or nontoxic; hence it is listed as an essential element for good health as well as a nutrition purpose for many organisms [13, 14]. Recently, Cr(III) oxidation to Cr(V) and/or Cr(VI) in biological systems came into consideration as a possible reason of antidiabetic activities of some Cr(III) complexes, as well as of long-term toxicities of such complexes. The specific interactions of Cr(III) ions with cellular insulin receptors are caused by intra- or extracellular oxidations of Cr(III) to Cr(V) and/or Cr(VI) compounds, which act as protein tyrosine phosphatase (PTP) inhibitors [15]. Chromium is also essential for the metabolism of higher animals; for example, impaired carbohydrate metabolism seen in Cr-deficient humans can be corrected by administration of small amounts of the metal. Cr(III) is identified and partially characterized as the glucose tolerance factor (GTF) believed to be essential for the normal disposition of glucose loads [16]. Hence, due to brilliant properties of both morin and Cr(III), it was considered worth to carry out their interaction as represented in Scheme 1.

## 2. Experimental

### 2.1. Materials

All the reagents and solvents were of analytical or chemically pure grade. Morin hydrate and DPPH^•^ (2,2′-diphenyl-1-picrylhydrazyl) were purchased from Sigma (St. Louis, MO, USA). Chromium(III) chloride, sodium *di*-hydrogen phosphate, and *di*-sodium hydrogen phosphate were obtained from Merck and HPLC grade methanol from Fisher scientific Ltd. (Leicestershire, UK), KBr from Aldrich Chemical Co. (Taufkirchen, Germany), and potassium ferricyanide and trichloroacetic acid were purchased from Fluka (Buchs, Switzerland), whereas the ferric chloride was obtained from Acros Organics (Belgium, USA). All the reagents were weighed with an accuracy of ±0.0001 g.

### 2.2. Physical Measurements

UV-Vis spectra were obtained by Perkins Elmer Lambda 35 UV-Vis double beam spectrophotometer, using standard 1.00 cm quartz cells in methanol solvent. The FT-IR spectra were recorded in the spectral range 4000–400 cm^−1^ on a Thermo Scientific Nicolet iS10 FT-IR instrument by KBr disc method. ^1^H NMR spectra were recorded on a Bruker 500 MHz spectrometer in DMSO, using TMS as internal reference. TG/DTA curves were obtained by using the Pyris Diamond TG/DTA (Perkin-Elmer instrument technology by SII) under nitrogen at the heating rate of 10°C min^−1^ from ambient to 600°C. The DSC curves were obtained using DSC822^e^ Mettler Toledo at the heating rates of 20°C min^−1^ in aluminum crucible under nitrogen atmosphere, respectively.

## 3. Synthesis

### 3.1. Synthesis of Complex

In a 50 mL two-necked round-bottomed flask equipped with electromagnetic stirrer and a thermometer, solid morin (0.6044 g or 0.2 mmol) was dissolved in MeOH and stirred until it was completely dissolved within 15 minutes and the solution became clear yellow colored and then we quickly added solid CrCl_3_·6H_2_O salt (0.266 g, 0.1 mmol) into the flask. The content was heated at 85°C due to relative kinetic inertness of chromium chloride and then refluxed the contents at least for 8 hours; the color of solution was changed to dark brown. The solution was filtered and run on rotary evaporator to remove the solvent. The compound was scratched and washed with the diethyl ether solvent to remove the impurities. Finally, the complex compound was dried in a vacuum oven and obtained in 79% yield. Elemental analysis found C, 43.60; H, 4.45%. Anal. Cal. for [Cr(C_15_H_9_O_7_)_2_(H_2_O)_2_]Cl·2H_2_O: C, 43.20; H, 4.11%, respectively. It was soluble in H_2_O, MeOH, EtOH, DMSO, DMF, acetone, and* n*-hexane and completely insoluble in DCM and chloroform.

### 3.2. Antioxidant Activity

The antioxidant activity for morin and the synthesized complex of Cr(III)-morin was studied spectrophotometrically by DPPH• method. DPPH• itself is a stable commercially available free radical. Its solution in methanol is violet upon reduction by an antioxidant it changes to corresponding yellow color. The absorbance of DPPH• is noted at 515 nm, which decreases by addition of an antioxidant sample. For the measurement of relative antioxidant activity of both of the compounds, they were prepared of appropriate concentration in methanol [17]. Their concentration was fixed for 0.2 mg/mL and thereof almost 50 *μ*L of each solution was mixed with 2 mL of the solution of DPPH• free radical having concentration of 0.1 mM. The decline in absorbance of DPPH• solution made by a sample compound was noted with the gape of every 5 minutes in the order of 0, 5, 10, 15, 20, 25, and 30 minutes till reaching the steady state. Finally, we calculated the percent scavenging (SC%) for each sample by using formula (1) and compared the inhibitory activity of both samples for their antioxidant potential [18]:

(1) $$\begin{array}{c} \text{SC}\%=\frac{\text{Absorbance}\;\text{of}\;\text{control}-\text{Absorbance}\;\text{of}\;\text{sample}}{\text{Absorbance}\;\text{of}\;\text{control}}\times 100. \end{array}$$

### 3.3. Ferric Reducing Antioxidant Power

Ferric reducing power of morin and the Cr(III)-morin complex was also studied by using the method of Oyaizu [19]. In this method, the absorbance of the various concentrations of the samples, that is, 0.5, 1.0, 1.5, and 2.0 mg/mL, was measured at 700 nm against the blank, containing all the reagents except the sample ones. The experimental procedure was worked out by adding 0.5 mL portion from sample solutions to the 2.5 mL phosphate buffer of 0.2 M concentration (6.6 pH); subsequently this solution was added to 2.5 mL of 1% solution of potassium ferricyanide. The resulting solutions of both of the compounds were applied for incubation under the temperature of 50°C for 20 minutes duration. Then to stop the reaction, 2.5 mL of 10% solution trichloroacetic acid was added. The solutions were centrifuged with the 3000 rpm speed for 10 minutes length of time. After that, 2.5 mL from upper layer of sample solutions was mixed up with 2.5 mL and 0.5 mL distilled water and ferric chloride (0.1%), respectively. Lastly, we measured the absorbance of the sample solutions and compared them for their reducing potential. A graph of absorbance versus sample concentration was plotted to observe the reducing power [18].

## 4. Results and Discussion

### 4.1. UV-Vis Study

**Figure 2:**
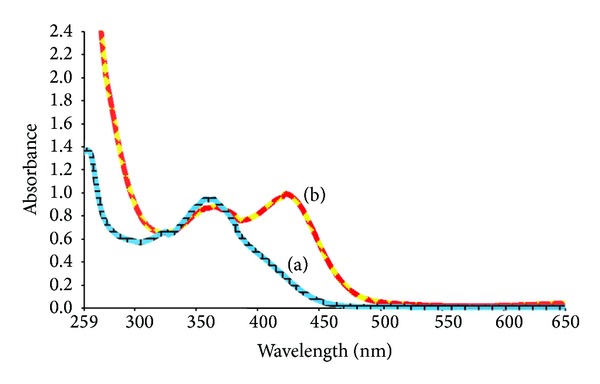
Relative UV-Vis spectra of morin and Cr(III) complex in DMSO. (a) UV-Vis spectrum of morin; (b) UV-Vis spectrum of morin-Cr(III) complex.

Morin is one of the important members of flavonoid family which exhibits two distinct peaks in the electronic spectrum, where peak I appears at 359 nm for cinnamoyl part and simultaneously band II emerges at 263 nm for benzoyl part [20] by analyzing in DMSO solvent. In fact, morin possesses various hydroxyl groups to form metal complexes in all three rings: hydroxyl groups on B ring, as well as the 3-hydroxy-4-keto and 5-hydroxy-4-keto groups on the A and C rings. The hydroxy-keto sites on the A and C rings are of particular interest because the 3-hydroxy and the 5-hydroxy sites are in competition for metal binding and it is disputed at which of these positions metal binding is predominant. Discrepancies also exist in reports of the preferred metal chelation site on the A and C rings of morin [21]. From the three potential chelating sites of morin, it is very important to decide which site chelates to the Cr(III) metal ion. Typically the ligand molecule would utilize its most suitable chelation site to form more stable complex. However, hydroxy-keto sites form a highly stable complex because it creates an additional ring with extended conjugation and more delocalization resulting in the formation of more stable complex and form a big pi bond system, whereas an additional chelation site between 2′-hydroxyl group of ring B and 3-hydroxyl group of ring C, which likely forms because of easy ionization of 2′-hydroxyl group, looks highly uncertain. It is because of the fact that this chelation site seems to be weak and relevant only in the absence of more potent chelation sites [22]. Moreover, the amount of bathochromic shift is also indicative of the involvement of certain number of chelating sites as well as the location of appropriate chelating site in morin. Thus, in the case of Cr(III), it would show significant bathochromic shifts perhaps due to high charge density and consequently strong bonding with the ligand molecule. The coordination of Cr(III) with morin caused the red shift of 101 nm in band II, while band I shows very small red shift of 64 nm as illustrated in Figure 2. Thus the chelation of Cr(III) ion caused significant shift in both of the bands (from 359 nm to 423 nm for band I and from 263 nm to 364 nm for band II). In consequence, the red shift of band II is higher than that of band I. It indicates that chelation shows more conjugation at benzoyl system in A ring instead of cinnamoyl system in B ring. Thus it can be concluded from the study that deprotonation of 5-OH takes place at the time of complex formation rather than 3-OH to form morin-Cr(III) complex [23].

However, evidence for metal binding at the 5-hydroxy-4-keto position in morin is also substantial such as Ce(III) and Gd(III) bind morin at the 5-hydroxy-4-keto position^31^. In addition, structurally similar quercetin was also found to preferentially bind Fe(III) at 5-hydroxy-4-keto site studied by UV-Vis and cyclic voltammetry [24] and chelation of Cr(III) with quercetin has also been studied to occur at 5-hydroxy-4-keto position [23]. Morin also binds Fe(II) at the 5-hydroxy-4-keto site but the conclusion is based on DPPH• and superoxide radical scavenging assays and not on direct measurements of metal binding [25]. In addition, rutin is also reported to chelate through 5-hydroxy-4-keto site with TiO(C_2_O_4_)_2_^2−^ [26].

### 4.2. FTIR and NMR Spectra

**Figure 3:**
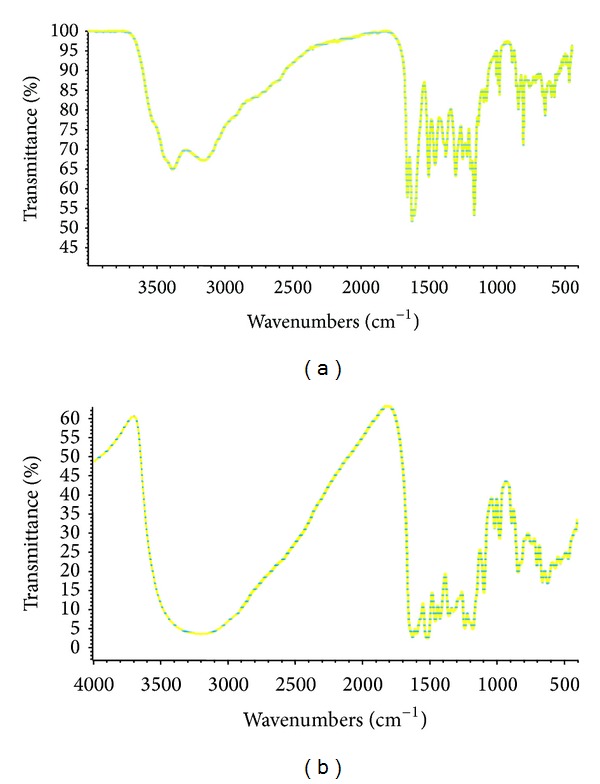
IR spectra of (a) morin and (b) Cr(III)-morin complex illustrating the major changes observed between them.

IR spectra provide very important information about the complex structure (Figure 3). Comparing the spectral data of ligand molecule with its corresponding Cr(III)-morin complex may provide the idea about such peaks which either have newly formed or disappeared or even changed their position after complexation. But the regions where major changes have emerged are well described here. The spectra for both of the compounds were recorded in 4000–400 cm^−1^ range. A broad absorption band can be easily viewed at 3000–3400 cm^−1^ as a big bound in the complex and morin ligand molecule may show the presence of OH/H_2_O stretching vibrations [27]. Simultaneously, a sharp peak observed in the spectrum of morin molecule at 1662 cm^−1^ has been assigned to the stretching vibrations of carbonyl (C=O) function located at 4-position. This carbonyl group in complex shows remarkable difference in frequency (Δ*ν* = 39 cm^−1^) form the morin molecule and appears at 1623 cm^−1^. It denotes that the morin molecule may coordinate the metal ion from carbonyl position. In order to decide which hydrogen, either 3-OH, or 5-OH has been replaced to bond the metal ion in conjunction with carbonyl may become clear from ^1^H NMR study. In addition, there is no major change observed in the frequency of *ν*(C–O–C) and *ν*(C=C) that appear at 1310 cm^−1^ and 1613 cm^−1^ in morin and at 1320 cm^−1^ and 1594 cm^−1^ in the complex perhaps because the ring oxygen is not involved in complexation process [28]. The most important peak appears around a very low frequency value of 466 cm^−1^ due to the formation of Cr(III)-O bond, indicating that the metal ion has become the part of giant morin molecule and confirms the formation of complex structure, because this peak is not present in the spectrum of morin molecule [29]. However, the interaction of metal ion to occur at 3-hydroxy or 5-hydroxy group cannot be clearly judged here. It becomes clear by undertaking the ^1^H NMR study of the ligand and complex compound.

^1^H-NMR study was carried out in DMSO for both morin molecule and complex structure of Cr(III)-morin to know which hydroxyl group proton out of five in morin, that is, 3-OH, 5-OH, 7-OH, 2′-OH, and 4′-OH, has been replaced in complexation. After coordination of Cr(III) metal ion, the protons of morin undergo the change in their chemical shift values either upfield or downfield due to increased conjugation. The signals in the morin spectrum are quite sharp but upon coordination they become broad. It was also observed that the signals in the morin spectrum appear at 9.40 ppm for 2′-OH and 4′-OH group protons, 9.74 ppm for 3-OH proton, 12.61 ppm for 5-OH, and 7-OH at 10.66 ppm values, respectively, whereas the Cr(III) complex shows only four visible signals except 5-OH proton signal but those four signals may appear with significant change in their chemical shift values due to complexation because it changes the environment of protons. Thus 3-OH, 7-OH, 2′-OH, and 4′-OH protons may appear in the complex at 9.81, 9.86, 6.42, and 7.23 ppm, respectively. Therefore, it becomes comprehensible that metal ion replaces the 5-OH proton that leads to the destruction of hydrogen bonds after chelation and morin acts as monobasic bidentate ligand. Therefore, ^1^H-NMR study provides very important information regarding the complex structure that 5-OH group is main site which is involved in complex formation in conjunction with 4C=O, indicating that the whole molecule retains the planar structure of the natural morin [30, 31]. A broad signal appeared at 3.45 ppm value represents the presence of water molecules in the complex structure [32]. Moreover, the data of the chemical shifts have been given in Table 1.

It was observed from the relative shift in ^1^H NMR signals of Cr(III) complex to the free ligand precursor that the lack of free ligand precursor peak and observed shifts in the spectrum conclusively indicates the binding of the ligand to the metal ion. The 5-OH proton signal was not observed in the spectrum of the complex during the complexation process because this phenolic group participates in coordination to the metal ion. The differences in the relative broadening reflect the different metal-proton distances, the broader peaks being associated with the proton nuclei of the ligand closer to the metal ion and these broad resonances support the octahedral geometry of the complex. These observances suggest that aromatic ring coordinates to the metal ion through phenolic and carbonyl groups [33].

## 5. Thermal Study

### 5.1. TG-DTA and DSC Study

The study shows that there are three distinct breaks in the thermogram of the complex structure. Dehydration as well as decomposition of the complex takes place at different temperatures. At the first step, the endothermic dehydration of crystalline water molecules takes place at the temperature of 80–100°C with the weight loss of 4.45%, while at the second step, weight loss of 4.20% pertains to the dehydration of coordinating water molecules at the temperature range of 170–210°C. Finally, the exothermic decomposition of morin molecules as third step starts from the temperature of 310°C and continues onward. The weight loss was found to be about 70.55% for this step. The residue of metal atom remains behind because under nitrogen atmosphere no metal oxide will be formed. DSC thermogram is also in complete harmony with the TG-DAT results. It shows the removal of crystalline water molecules at 110°C whereas coordinating water molecules at 230°C. A very sharp peak was also observed for melting point at 389°C, while the removal of morin molecules takes place at above 450°C [34].

**Figure 4:**
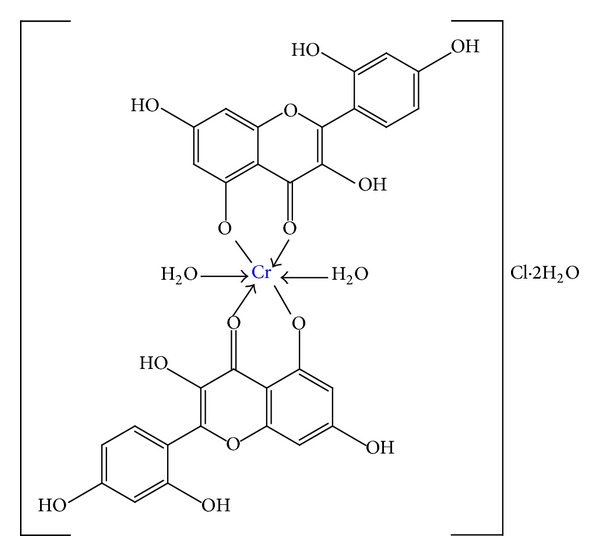
Tentative structure of Cr(III)-morin complex.

Further the structural formula of the Cr(III)-morin complex consistent with the results obtained is illustrated in Figure 4. Since, for steric reasons, the complex usually includes no more than two morin molecules, the degree of complex formation also depends upon the solvent system employed; in general, methanol favored the formation of the 1 : 2 dimer, whereas dimeric complex may be suppressed in other solvents [31, 35]. In fact, the metal cation often reacts in the form of aquo complex with some coordinating water molecules. Accordingly, it is very necessary to investigate the interaction between morin and hydrated Cr(III) ion to make clear whether the existence of water molecules in Cr(III) ion has any effect on the complex structure. Since chromium atom has six coordination sites, so two water molecules may be easily added by the Cr(III)-morin complex [23].

## 6. Antioxidant Activity

### 6.1. DPPH• Radical Scavenging Activity

**Figure 5:**
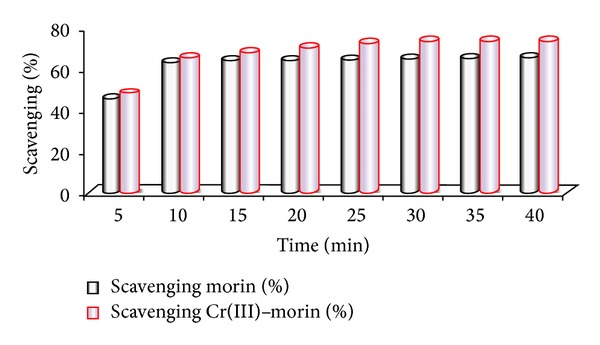
Relative antioxidative potential of morin and Cr(III)-morin complex.

**Scheme 2:**
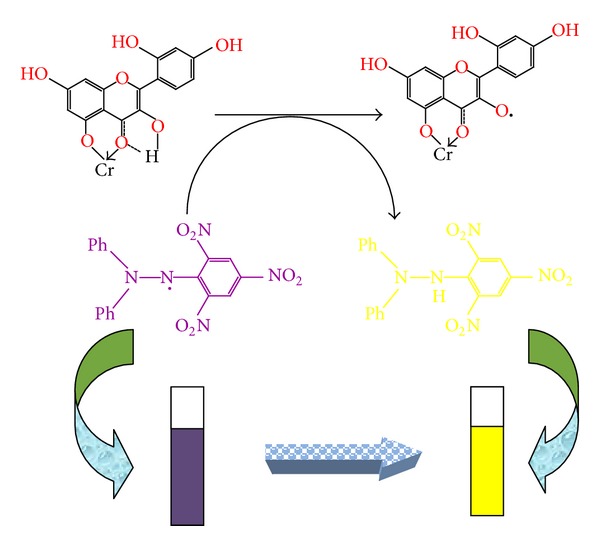
The addition of sample compound to the DPPH• may change its color from violet to yellow (also shown as cuvettes) because the proton from the sample is transferred to DPPH• by converting it into corresponding hydrazine form.

Morin is shown to be a potent scavenger of DPPH• and ABTS• free radicals [36, 37]. Despite the absence of catechol (*o*-dihydroxy) structure of the B ring which is recognised as the main prerequisite for high radical scavenging potency [38], the activity of morin is comparable to flavonoids with 3′,4′-dihydroxy moiety on the B ring. It is also reported by hydrogen atom transfer (HAT), single electron transfer followed by proton transfer (SET-PT) and sequential proton loss electron transfer (SPLET) mechanisms of morin, that 3-OH group is the most favored site for homolytic O–H breaking; on the other hand 5-OH group is not involved in the antioxidant mechanism; the main reason lying behind this is that the 5-OH group forms a strong H-bonding with O4 atom in parent molecule and 7-OH group is also preferred in radical inactivation. Thus, antiradical activity of morin is related to its ability to transfer the phenolic H-atom (proton together with one of its two bonding electrons) to free radical by producing phenoxyl radical. Hence, to be an effective phenoxyl free radical, it should react the substrate slowly but rapidly with DPPH• [39]. In addition to the radical scavenging, the metal binding is also attributed to the antioxidant activities. At the same time, the multiple binding sites in morin contribute to their strong antioxidant properties [40]. Generally, by chelating metal ions, morin prevents metal-catalyzed free radical generation and their subsequent reactions and accordingly protects very important biologically active molecules from oxidative stress [41]. Thus chelated Cr(III)-morin complex is more effective free radical scavenger than the corresponding free morin due to the acquisition of additional superoxide dismutating centers (Figure 5) [42]. However, upon addition of sample compound, the violet color of DPPH• changes into yellow color (shown in cuvettes) because the proton from the sample is transferred to DPPH• and converts it into corresponding hydrazine form as given in Scheme 2. Thus, the study may be useful to prevent oxidative damage because in body many free radicals are generated to produce age-related diseases such as aging, cancer, and cardiovascular and neurodegenerative diseases. Hence, to prevent the free radical damage, it is necessary to administer such drugs or herbals that may be rich in antioxidants like morin. That is why there is need to produce and synthesize the antioxidants with remarkable or improved effects that may be more useful and effective drugs than earlier existing ones.

### 6.2. Ferric Reducing Mechanism

**Figure 6:**
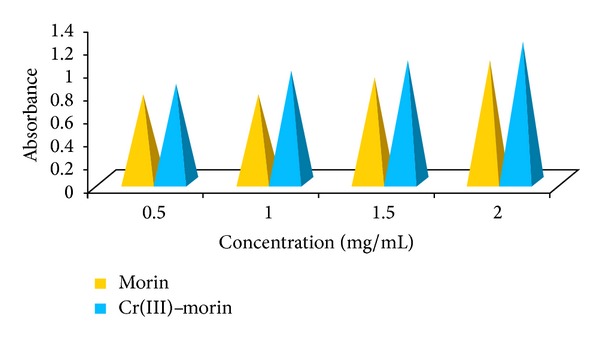
Ferric reducing potential of morin and its Cr(III)-morin complex.

The reducing potential of morin and morin-Cr(III) complex was determined. It acts as an indicator of sample's potential antioxidant power. The higher reducing power of the sample is correlated with its higher absorbance. The reducing power of methanolic sample solutions was measured as a function of their concentration, as illustrated in Figure 6.

**Scheme 3:**
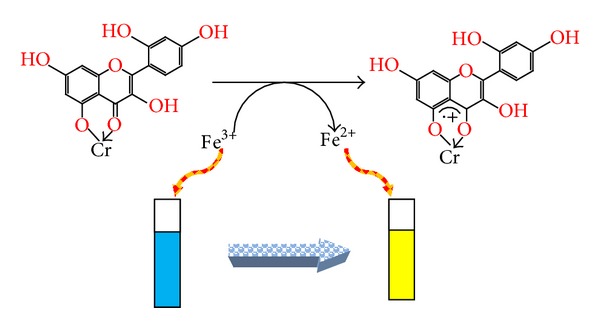
The mechanism shows that the blue color of the antioxidant samples (the reduced product leading to the formation of intense Perl's Prussian blue Fe_4_[Fe(CN)_6_]_3_ complex which is reduced to Fe^2+^) may change into yellow color upon addition of free Fe^3+^ ions (as illustrated through cuvettes).

Absorbance of samples increased with increase in their concentrations. The higher reducing capacity originates from the higher amounts of polyphenolics (reductones) present and therefrom the antioxidant potential of the investigated compounds can be measured [43]. The reducing power was found to be maximum for complex that goes to about the absorbance of 1.22 at 2 mg/mL of concentration, whereas morin shows relatively less reducing potential and there is a direct correlation between antioxidant activity and reducing power of samples. The reducing properties are generally associated with the presence of reductones, which have been shown to exert antioxidant action by breaking the free radical chain. The presence of reductants (that is antioxidants) causes the reduction of Fe^3+^/ferricyanide complex to the ferrous form that can be monitored by measuring the formation of Perl's Prussian blue at 700 nm [44]. Thus complex shows the highest reducing power than morin because it contains the highest amounts of reductones and polyphenolics than morin. In this assay, yellow color of the test solution changes to green or blue color depending on the reducing power of antioxidant samples. Addition of free Fe^3+^ to the reduced product leads to the formation of the intense Perl's Prussian blue Fe_4_[Fe(CN)_6_]_3_ complex which is reduced to Fe^2+^ and changed into yellow color (cuvettes) as shown in Scheme 3 [45, 46].

At the same time, the study proves that the complexation between morin and metal ion takes place, which may also be very useful to remove many toxic metal ions from body at specific conditions through metal chelation therapy and it can also be useful to supply the essential metal ions to body if it is deficient to any essential metal ion.

## 7. Conclusion

It has been concluded from the study that Cr(III) forms complex from 4C=O and 5-OH groups, which is proved from various techniques such as UV-Vis, IR, and ^1^H NMR studies. From DPPH radical scavenging as well as ferric reducing power studies, it has been proved that complex of Cr (III)-morin is a more powerful antioxidant than the morin molecule alone. In addition, the study shows very nice impact for designing anticancer drugs, combined with their potential cytotoxic and antioxidant activities, which can be targeted selectively against cancer cells and increase their therapeutic index and additional advantages over other anticancer drugs.

## Figures and Tables

**Table 1:** ^
1^H NMR data for morin and Cr(III)-morin complex.

H	Morin *δ*, ppm	Cr(III)-morin *δ*, ppm
2′-OH	9.40	6.42
4′-OH	9.40	7.23
3-OH	9.74	9.81
5-OH	12.61	—
7-OH	10.66	9.86
